# An Optimized Model Based on Deep Learning and Gated Recurrent Unit for COVID-19 Death Prediction

**DOI:** 10.3390/biomimetics8070552

**Published:** 2023-11-17

**Authors:** Zahraa Tarek, Mahmoud Y. Shams, S. K. Towfek, Hend K. Alkahtani, Abdelhameed Ibrahim, Abdelaziz A. Abdelhamid, Marwa M. Eid, Nima Khodadadi, Laith Abualigah, Doaa Sami Khafaga, Ahmed M. Elshewey

**Affiliations:** 1 Computer Science Department, Faculty of Computers and Information, Mansoura University, Mansoura 35561, Egypt; zahraatarek@mans.edu.eg; 2 Faculty of Artificial Intelligence, Kafrelsheikh University, Kafrelsheikh 33516, Egypt; mahmoud.yasin@ai.kfs.edu.eg; 3 Computer Science and Intelligent Systems Research Center, Blacksburg, VA 24060, USA; sktowfek@jcsis.org; 4 Department of Communications and Electronics, Delta Higher Institute of Engineering and Technology, Mansoura 35111, Egypt; mmm@ieee.org; 5 Department of Information Systems, College of Computer and Information Sciences, Princess Nourah Bint Abdulrahman University, P.O. Box 84428, Riyadh 11671, Saudi Arabia; 6 Computer Engineering and Control Systems Department, Faculty of Engineering, Mansoura University, Mansoura 35516, Egypt; 7 Department of Computer Science, Faculty of Computer and Information Sciences, Ain Shams University, Cairo 11566, Egypt; abdelaziz@cis.asu.edu.eg; 8 Department of Computer Science, College of Computing and Information Technology, Shaqra University, Shaqra 11961, Saudi Arabia; 9 Faculty of Artificial Intelligence, Delta University for Science and Technology, Mansoura 35712, Egypt; 10 Department of Civil and Architectural Engineering, University of Miami, 1251 Memorial Drive, Coral Gables, FL 33146, USA; nima.khodadadi@miami.edu; 11 Computer Science Department, Al al-Bayt University, Mafraq 25113, Jordan; aligah.2020@gmail.com; 12 College of Engineering, Yuan Ze University, Taoyuan 32003, Taiwan; 13 Hourani Center for Applied Scientific Research, Al-Ahliyya Amman University, Amman 19328, Jordan; 14 MEU Research Unit, Middle East University, Amman 11831, Jordan; 15 Department of Electrical and Computer Engineering, Lebanese American University, Byblos 13-5053, Lebanon; 16 School of Computer Sciences, Universiti Sains Malaysia, Pulau Pinang 11800, Malaysia; 17 School of Engineering and Technology, Sunway University Malaysia, Petaling Jaya 27500, Malaysia; 18 Department of Computer Sciences, College of Computer and Information Sciences, Princess Nourah Bint Abdulrahman University, P.O. Box 84428, Riyadh 11671, Saudi Arabia; dskhafga@pnu.edu.sa; 19 Computer Science Department, Faculty of Computers and Information, Suez University, Suez 43512, Egypt; elshewy86@gmail.com

**Keywords:** machine learning, convolutional neural network (CNN), gated recurrent unit (GRU), Internet of Medical Things (IoMT), COVID-19 pandemic, death prediction

## Abstract

The COVID-19 epidemic poses a worldwide threat that transcends provincial, philosophical, spiritual, radical, social, and educational borders. By using a connected network, a healthcare system with the Internet of Things (IoT) functionality can effectively monitor COVID-19 cases. IoT helps a COVID-19 patient recognize symptoms and receive better therapy more quickly. A critical component in measuring, evaluating, and diagnosing the risk of infection is artificial intelligence (AI). It can be used to anticipate cases and forecast the alternate incidences number, retrieved instances, and injuries. In the context of COVID-19, IoT technologies are employed in specific patient monitoring and diagnosing processes to reduce COVID-19 exposure to others. This work uses an Indian dataset to create an enhanced convolutional neural network with a gated recurrent unit (CNN-GRU) model for COVID-19 death prediction via IoT. The data were also subjected to data normalization and data imputation. The 4692 cases and eight characteristics in the dataset were utilized in this research. The performance of the CNN-GRU model for COVID-19 death prediction was assessed using five evaluation metrics, including median absolute error (MedAE), mean absolute error (MAE), root mean squared error (RMSE), mean square error (MSE), and coefficient of determination (R^2^). ANOVA and Wilcoxon signed-rank tests were used to determine the statistical significance of the presented model. The experimental findings showed that the CNN-GRU model outperformed other models regarding COVID-19 death prediction.

## 1. Introduction

Presently, there are many diseases that have become prevalent [1]. The COVID-19 coronavirus illness was initially identified in December 2019 in China, in Wuhan, and has since spread around the world. The virus spreads quickly because it is easily transmitted from one individual to another [2]. Technology and science play a significant part in this confusing conflict. China focused on medical research and using robots to deliver food and medical supplies, automatons to clean up open spaces, and broadcasting and watching sound information exchange publicly to encourage people to remain at home. In order to help COVID-19 patients, a lot of human expertise was employed to detect the new particles in transit [3]. Numerous studies are being conducted to monitor, trace contacts, forecast, and diagnose the COVID-19 illness. One of these innovations is the Internet of Things (IoT), which is attracting international attention for its growing role in healthcare systems’ ability to forecast, identify, prevent, and monitor the majority of infectious illnesses. Similar to how it aids in the battle against COVID-19, it also helps in the detection of the COVID-19 epidemic through monitoring, contract tracking, and connecting with IoT-based efficient technologies [4]. IoT is a network of connected electronic devices, actuators, sensors, and data that are collected in their raw form and delivered online via the internet [5]. 

Healthcare represents one of the vital domains that employ IoT systems and smart devices for supervision. IoT is a successful field for many sectors and academic subjects. IoT transition supplies modern healthcare services with scientific and socioeconomic perspectives. Since the outbreak of the pandemic, several scientific groups have stepped up their efforts to employ a variety of methods to address this global issue. IoT techniques are utilized in certain procedures, such as prenatal screening, patient monitoring, and post-patient event response, to reduce COVID-19 exposure to other individuals [6]. The IoT-based healthcare system is described in depth by the Internet of Medical Things (IoMT) [7]. When employed during this epidemic, the IoMT can assist patients in receiving appropriate medical treatment at home, and healthcare officials and governments can utilize the extensive dataset built for COVID-19 spread control. People with mild symptoms can buy diagnostic and medical equipment, including thermometers, smart watches, smart helmets, drugs, protective masks, and tracking infection packages. Patients can routinely upload overall health records via a wireless network and the internet to medical cloud servers, and these data can be sent to the closest hospitals, health centers, or clinics, as well as the Centers for Disease Control (CDC) [8]. IoMT offers a platform for smart devices and sensors to communicate effectively in a smart environment, and makes it simple to interchange data and information online.

A critical component in measuring, evaluating, and diagnosing diseases is artificial intelligence (AI). It can be used to anticipate cases as well as forecast the number of alternate incidences, recovered cases, and injuries, along with specific software engineering analyzers that concentrate on the detection of patients through the production of medical images like CT filters and X beams; a lot of professionals employ AI to uncover novel drugs and treatments [3]. AI is made to act and think like a human brain, automating many tasks by imitating its thought processes. In preparation for COVID-19’s eventual cross-country accessibility, machine learning (ML) and deep learning (DL) techniques can be utilized to track typical behavior using open data sources from real-time applications. These techniques can forecast the immediate future and aid in minimizing the negative impacts of COVID-19 [9,10,11]. Around the world, concerns have been raised about the COVID-19 pandemic strategy’s capabilities and delivery, quick response, linked information, and evaluation [6]. Even though the current deep learning techniques have considerably improved their performance for COVID-19 detection, the bulk of these techniques still have overfitting issues [12]. Advanced healthcare informatics and computational intelligence are enabling the development of secure and patient-oriented IoT systems that use BiLSTM deep learning and decision tree models to support automated diagnosis [13].

Optimization is a powerful tool used in various domains, and it plays a significant role in the medical field. Optimization aims to achieve the best possible outcomes or decisions under specific conditions based on a set of variables and defined criteria. In the medical domain, optimization is applied in diverse areas, such as the prediction and classification of monkeypox disease [14,15,16], feature selection and classification in diagnostic breast cancer [17], classification of diabetes [18], neurodegenerative disorders [19], and classification of COVID-19 in chest X-ray images [20]. The use of optimization in the medical field contributes to enhancing patient outcomes and maximizing the utilization of available resources.

An IoT-based system is necessary to address the monitoring and diagnostic issues, as it will aid in implementing stay-at-home protocols and decreasing the number of medical resources required [19]. With this method, information on healthcare facilities can be gathered, allowing for more efficient medical care to be established and more equitable distribution of government and private donations of medical supplies and equipment to hospitals and clinics [20]. In order to provide timely and effective medical services, especially in light of COVID-19, the disciplines of IoT and AI have been forcefully urged to routinely automate and simplify numerous duties for health professionals. This research delves into the role that IoMT and AI will play in bringing healthcare to a completely new level in the face of the COVID-19 epidemic. The hybrid deep learning model of a convolutional neural network with a gated recurrent unit for predicting COVID-19 mortality via the IoT can be combined with a biosensor for real-time patient monitoring. A biosensor is a device that detects and measures biological, chemical, or physical signals in the body. By integrating the biosensor with the hybrid CNN-GRU model, the system can collect continuous and accurate data to improve the accuracy of COVID-19 mortality prediction. This can lead to better patient outcomes and more efficient resource allocation in clinical settings. Monitoring patients remotely and continuously, predicting the risk of COVID-19 complications and mortality, and developing new and personalized treatment strategies are the main motivations of this study. 

This paper used an Indian dataset to test the efficacy of a convolutional neural network (CNN) equipped with a gated recurrent unit (GRU) for predicting the next round of the COVID-19 pandemic. In all, there are 4692 examples and eight characteristics in the dataset utilized for this research. The CNN-GRU model for COVID-19 death prediction is calculated using five assessment metrics: root mean squared error (RMSE), mean square error (MSE), median absolute error (MedAE), mean absolute error (MAE), and coefficient of determination (R^2^). The main contributions of this study are as follows:

- The research utilizes ML models for the death prediction of COVID-19 based on an Indian dataset from Kaggle; it includes eight features and 4692 instances.
- Data preprocessing was performed on the chosen dataset using data normalization and mean imputation.
- A novel CNN-GRU model with an IoT-based framework is suggested for predicting suspected cases of the COVID-19 pandemic.
- The proposed model (CNN-GRU) and some of the ML models (Random Forest (RF) Regressor, Support Vector Regressor (SVR), K-Nearest Neighbor (KNN) Regressor, Bayesian Ridge (BR) Regressor, Gradient Boosting (GB) Regressor, and Dummy Regressor (DR)) are examined and compared.
- The evaluation of the proposed approach was applied using MAE, MedAE, MSE, R2, and RMSE. The obtained results illustrated that the CNN-GRU model performed better than other prediction models and several studies.
- ANOVA and Wilcoxon signed-rank tests are used to determine if model performance differences are statistically significant.

The remainder of the paper is structured as follows. Related research to the search problem is summarized in Section 2. The experimental setup for the suggested ML techniques via IoT is covered in Section 3. Section 4 discusses the arguments for the findings and main results. Section 5 concludes with a summary of our findings.

## 2. Related Work 

The use of biosensors and the Internet of Medical Things (IoMT) in COVID-19 prediction is an active area of research. Biosensors can detect biological signals and transmit data in real-time to IoMT-enabled devices, allowing for the continuous monitoring of patients [21]. These data can be used in combination with machine learning models to predict disease progression, severity, and mortality. Several studies have investigated the use of biosensors and IoMT in COVID-19 prediction. For example, a recent study developed a biosensor-based system that uses artificial intelligence algorithms to predict COVID-19 severity and mortality. The system integrates biosensors with IoMT-enabled devices, allowing real-time patient monitoring and data collection [22].

Another study used a wearable biosensor to monitor COVID-19 patients and predict disease severity based on changes in heart rate variability [23]. The study found that changes in heart rate variability were associated with disease severity and could be used to predict disease progression. Overall, the integration of biosensors and IoMT has the potential to improve COVID-19 prediction, leading to better patient outcomes and a more efficient allocation of healthcare resources [24]. Healthcare is being revolutionized by cutting-edge innovations like IoT and smart sensors, robots, artificial intelligence (AI), blockchain, machine learning (ML), augmented reality (AR), virtual reality (VR), big data, cloud computing, drones and intelligent mobile applications, 5G, and so on. Pre-screening, early identification, monitoring quarantined/infected persons, estimating future infection rates, and other methods of dealing with COVID-19 were discussed. Research opportunities made possible by the deployment of cutting-edge technology to combat the COVID-19 pandemic are also explored [25,26,27]. A developed neural network model presented by Wieczorek et al. [28] that showed the spread of the COVID-19 virus using the NAdam optimizer achieved 99.00% accuracy. 

A six-tiered architecture of IoT tools for controlling the deadly COVID-19 virus was presented by Farhana Ajaz et al. [29]. The function of machine learning strategies in the identification of COVID-19 was explored. The effects of COVID-19 were mitigated in a number of ways, some of which made use of IoT technology. In addition, IoT could be applied in the medical field to guarantee people’s safety and health while keeping expenses down. Mir et al. [30] presented a real-time IoT-enabled architecture for COVID-19 diagnosis and prediction by gathering symptomatic indicators and better evaluating the virus’s characteristics. By mining health information acquired in real-time detection from sensing devices and IoT objects, the framework was able to determine the existence of the COVID-19 virus. The framework’s four primary parts were the data collection hub, the data analytics hub, the diagnostics hub, and the cloud system. This paper offered five machine learning methods for real-time pointing and detection of COVID-19 suspects. Results indicated an accuracy of 95% or higher using the applied machine learning methods.

Anita S. Kini et al. [31] developed a system for the screening of possible instances of COVID-19 using an ensemble of DL models and the IoT. The ensemble was made up of three common pre-trained DL models. The CT scans were collected using clinical IoT devices, and the automated diagnoses were processed by IoT platforms. Over the course of a four-class dataset, the proposed methodology was evaluated against 13 competing models. From their experiments, the suggested ensembled DL technique achieved a 98.98% success rate. As a result, the suggested methodology accelerated the process of identifying COVID-19. Fatema Al-Dhaen et al. [32] developed a simulation to study how ethical AI could help the advancement of IoMT in medical settings. Asghari et al. [33] suggested an IoT-based prediction method for colorectal cancer (CRC). Through the use of wearable embedded devices and healthcare IoT devices, it generates a CRC prediction technique by collecting vital clinical data via IoMT sensors and devices, enabling the medical staff to track the biomarkers of an aging individual over time.

To easily recognize the COVID-19 CT images and chest X-rays available to the public, a hybrid framework of the artificial neural network with parameters optimized using the butterfly optimization algorithm has been suggested and compared to the pre-trained GoogLeNet, AlexNet, and the SVM for COVID-19 recognition. With average accuracy of 90.48, 86.76, 84.97%, and 81.09 for the proposed model, AlexNet, GoogLeNet, and SVM, the experimental findings validated the effectiveness of the suggested model [34]. Khan et al. [35] suggested two novel DL frameworks, Deep Boosted Hybrid Learning (DBHL) and Deep Hybrid Learning (DHL) for efficient COVID-19 identification in the X-ray database. On the radiologist-verified chest X-ray database, the suggested COVID-19 identification frameworks were compared against traditional CNNs. Experiments showed that the DBHL, which combined the feature spaces of two deep CNNs, achieved high levels of accuracy equal to 98.53%. 

Shawni Dutta et al. [36] suggested a technique for checking verification using the principles of DL neural networks. The framework integrated long short-term memory (LSTM) and gated recurrent unit (GRU) for training the database, and the outcomes of the predictions matched those made by clinical physicians. The predictions were checked against the source data using some metric that had been established. The experimental outcomes demonstrated the efficacy of the suggested method in producing appropriate outcomes in light of the serious illness epidemic. Soudeh Ghafouri et al. [37] integrated LSTM, recurrent neural network, multilayer perceptron, and adaptive neuro-fuzzy inference system. Researchers evaluated multiple machine learning strategies for their ability to foretell the spreading of COVID-19. These models integrated data from illnesses with comparable patterns to COVID-19, allowing for the discovery of learning indicators that affect differences in COVID-19 dissemination across different locations or populations, as well as the implementation of what-if scenarios based on those approaches. Thus, these techniques, if used in policymaking, would aid in the development of effective interventions and the avoidance of ineffective restraints. From the previous work, we can conclude that the CNN-GRU model proposed in the paper is novel in the following ways.

It uses an Indian dataset to train and test the model, which is different from most other CNN-GRU models for COVID-19 death prediction, which have been trained on datasets from other countries. It incorporates an IoMT-based framework into the model, which allows the model to learn from real-time data on COVID-19 cases and deaths. It is evaluated using a variety of metrics, including MAE, MedAE, MSE, R2, and RMSE, which provides a more comprehensive assessment of the model’s performance. Compared to other recent models for COVID-19 death prediction, the CNN-GRU model proposed in the paper has the following advantages:

- It achieves better prediction performance on the Indian dataset, which is a challenging dataset due to its high variability.
- It is more robust to noise and outliers in the data.
- It is more interpretable, meaning that it is easier to understand how the model makes predictions.

Here are some specific examples of how the CNN-GRU model could be used to improve COVID-19 response:

- The model could be used to predict the number of COVID-19 deaths in different regions and countries. This information could be used to allocate resources more effectively and to develop targeted interventions to reduce mortality.
- The model could be used to identify high-risk populations for COVID-19 mortality. This information could be used to develop targeted public health campaigns and to provide support to vulnerable individuals.
- The model could be used to predict the impact of different public health measures on COVID-19 mortality. This information could be used to inform decision-making about how to best control the pandemic.

The CNN-GRU model is still under development, but it has the potential to be a valuable tool for COVID-19 response. Furthermore, the CNN-GRU model proposed in the paper is a novel and promising approach for COVID-19 death prediction. It has the potential to be used to develop early warning systems for future waves of the pandemic and to inform public health decision-making.

## 3. The Proposed CNN-GRU Model 

**Figure 1:**
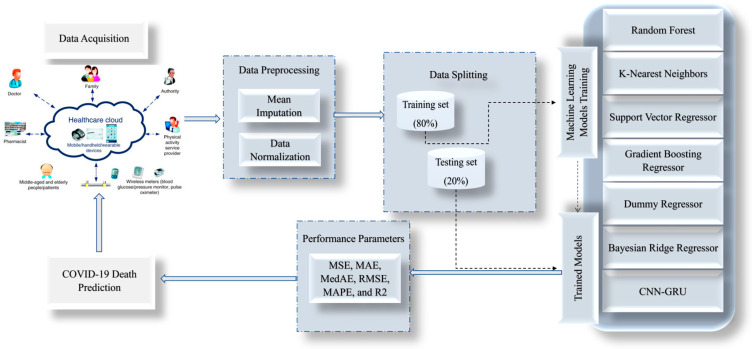
General structure of the proposed framework for the death prediction of the COVID-19 epidemic.

The proposed model is a hybrid model between CNN and GRU, utilized in this paper to predict COVID-19 via IoMT. The proposed model is compared with some ML models, such as Random Forest (RF) Regressor [38], K-Nearest (KNN) Regressor [39], Support Vector Regressor (SVR) [40], Gradient Boosting (GB) Regressor [41], Dummy Regressor (DR), and Bayesian Ridge (BR) Regressor [42], as shown in Figure 1. 

The CNN-GRU model for COVID-19 death detection was developed through a series of steps using preprocessed data collected from the Indian dataset. The first step involved preprocessing the data to ensure they were in a format the model could use. This involved cleaning the data, removing any missing values, and transforming them into a suitable form for analysis. The data are divided into 80% for training and the remaining 20% for testing. The next step was to apply a convolutional neural network (CNN) to the preprocessed data. The CNN extracted relevant features from the data, which could be used as input for the next step. Following the application of the CNN, a gated recurrent unit (GRU) was used to model the temporal relationships between the features extracted by the CNN. The GRU is a recurrent neural network capable of modeling time-series data, making it well-suited for this task. The output of the GRU was then fed into a fully connected layer, which was used to predict whether a patient was likely to die from COVID-19.

**Figure 2:**
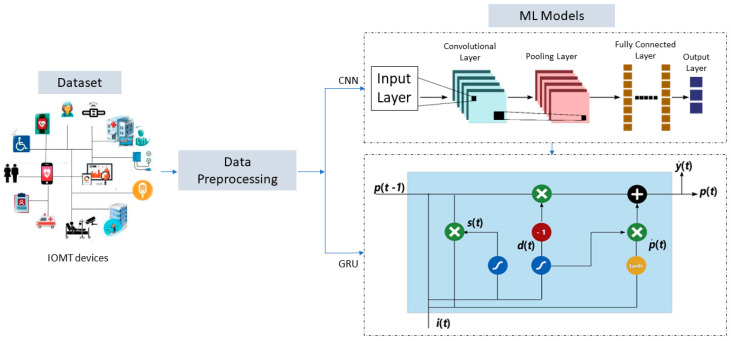
The steps of CNN-GRU for COVID-19 death detection for the preprocessed data.

The creation and implementation of these models were made possible by collecting and utilizing a comprehensive Indian dataset. To ensure the accuracy and reliability of the suggested algorithms, various validation techniques, such as mean squared error (MSE), mean absolute error (MAE), median absolute error (MedAE), root mean squared error (RMSE), and R-squared (R^2^), were employed. The proposed methodology is visually represented in Figure 2, outlining the steps taken to develop and evaluate the models. These rigorous testing and validation methods ensure that the models are dependable and accurate, making them valuable tools in various applications.

LSTM (long short-term memory) and GRU (gated recurrent unit) are both types of recurrent neural networks (RNNs) that are commonly used in time-series analysis and other applications that require the modeling of enrolled processed data. It was hypothesized that LSTM could solve the issue of vanishing and bursting gradients. In LSTM, “cells” are the fundamental data processing units [43]. It is possible to interpret these cells as advanced neuronal cells. Many gates in a cell regulate and keep open a pathway for data to go along for the duration of a potentially infinite series. Because of this capacity, LSTM can tell if data are relevant in the near and far future. Because of this, it works wonderfully for sequential problems of any kind. LSTM’s strength lies in its ability to store and transform the input cell memory into the output cell state via its cell state. According to the following equations, LSTM is made up of an input gate, a forget gate, an update gate, and an output gate [44,45]:

(1) $$i_{t}=\sigma \left(W_{i}\times \left[h_{t-1},x_{t}\right]+b_{i}\right)$$

(2) $$f_{t}=\sigma \left(W_{f}\times \left[h_{t-1},x_{t}\right]+b_{f}\right)$$

(3) $$c_{t}=tanh\left(W_{c}\times \left[h_{t-1},x_{t}\right]+b_{c}\right)$$

(4) $$o_{t}=\sigma \left(W_{o}\times \left[h_{t-1},x_{t}\right]+b_{o}\right)$$

(5) $$h_{t}=o_{t}\times tanh\left(c_{t}\right)$$

where $i_{t}$, $f_{t},\;c_{t},\;o_{t}$, and $h_{t}$ are the input, forget, update, output gate, and hidden layers.

CNN relies heavily on a supervised learning model inspired by how humans naturally pay attention to images. The convolutional neural network (CNN) is chosen over other machine learning models since it does not need any sort of feature extraction preparation [46]. The convolutional, max-pooling, and nonlinear activation layers make up a CNN, a category of deep neural networks. Convolution, the procedure that gives CNN its name, is carried out in the convolutional layer, which is regarded as a primary layer of the CNN. In the convolutional layer, the inputs to the layer are processed by means of kernels. Feature maps are created by convolving all the convolutional layer outputs [47]. Training a CNN model is difficult because of issues with disappearing and growing gradients. Two modern deep learning methods, GRU and LSTM, efficiently deal with this problem. To get around training issues and save the system’s current state across iterations, a new variant of CNN called the GRU has been created [48].

In 2014, Cho proposed a variation of the LSTM called the gated recurrent unit (GRU). Currently, GRU networks are mostly used for classification problems and are seldom used for regression problems. Given that there is no output gate in a standard LSTM, the training time is longer and more parameters are required [49]. The update gate in a GRU cell is a combination of the forget gate and the input gate, which is a concealed state. Not only that, but GRU unifies the hidden and cell states into a single state. These equations characterize the GRU’s hidden, updated, and reset states as follows [44]:

(6) $$p_{t}=\left(1-z_{t}\right)\times p_{t-1}+z_{t}\times p_{t}$$

(7) $$d_{t}=\sigma (W_{d}\times \left[p_{t-1},i_{t}\right])$$

(8) $$s_{t}=\sigma (W_{s}\times \left[p_{t-1},i_{t}\right])$$

(9) $$p_{t}=\tanh(W\times \left[{r_{t}\times p}_{t-1},i_{t}\right])$$

where $i_{t}$, $p_{t-1}$, $d,\;s_{t}$, and $p_{t}$ are the vectors of input, previous output, update gate, reset gate, and hidden layer, respectively. The parameter $z_{t}$ is a weighting factor that determines the extent to which the value of $p_{t}$ is influenced by the value of $p_{t-1}$.

While both LSTM and GRU networks have been shown to be effective in various tasks, the choice between them often depends on the specific problem being addressed, the size of the dataset, and the computational resources available. In some cases, LSTM networks can be preferable when modeling very long sequences, while GRUs can be a better choice for smaller datasets or when training time is a concern. Ultimately, the choice between LSTM and GRU networks should be made based on careful experimentation and the evaluation of their performance on the specific task at hand. Algorithm 1 outlines the steps involved in the proposed CNN-GRU model for COVID-19 death detection. The model is designed to take preprocessed data from the Indian dataset as input and generate predictions of the likelihood of COVID-19-related deaths in patients.
**Algorithm 1:** Proposed CNN-GRU for COVID-19 death prediction1.  **Input**: COVID-19 dataset D, Number of CNNs N. 2.  **Initialize** GRU parameters.     //Preprocess dataset 3.  **Normalize** sample in dataset D. 4.  **Divide** D into 2 subsets: training and testing. 5.   **Define** the CNN layer with filters, kernel size, activation function, and padding. 6.   **Apply** the CNN layer to the input data to extract relevant features. 7.   **Define** the GRU layer with hidden units, activation function, and dropout rate.     //Train CNNs 8.  **For**
*i* = 1 to *n* do 9.      **Train** CNN using the training set     //Build the GRU model 10.  **Add** the GRU layer of L1 units and set dropout = d1 and recurrent dropout = s1. 11.  **Compute** update gate *d_t_*, reset gate *s_t_* using Equations (7) and (8). 12.  **Compute** the candidate state *p_t_* using Equation (9) 13.   **While** stopping criteria did not met do 14.      **While** training for all instances do 15.          **Calculate** linear function as an activation function used in the output layer. 16.          **Update** weights and bias 17.      **End while** 18.   **End while**     //Test the proposed model 19.   **Test** hyperparameters with the test dataset. 20.   **Return** evaluate result in the test dataset.

## 4. Statistical Analysis of Dataset 

In this section, we utilized the standard Indian dataset available at Kaggle with the link https://www.kaggle.com/datasets/imdevskp/covid19-corona-virus-india-dataset, accessed on 6 November 2023.

The dataset collection on the COVID-19 outbreak in India includes various sources of information. The data collection began on January 30 when the first case of COVID-19 in India was reported, originating from China. The dataset includes multiple files, such as complete.csv, which provides day-to-day state-wise numbers of cases sourced from the Ministry of Health and Family Welfare (MoHFW) website. The dataset also includes information on the number of tests conducted on a daily basis and the latest state-level tests. The data sources for this collection are the Ministry of Health and Family Welfare, India’s official website, and the COVID-19 India Tracker website, with the data themselves accessible through the COVID-19 India Tracker Data API. These sources provide valuable information for tracking and analyzing the COVID-19 situation in India.

The dataset comprises 4692 instances and eight features, including latitude, longitude, total confirmed cases, cured/discharged/migrated, new cases, new deaths, and new recoveries, with the target feature being deaths. Since we have 4692 cases, 80% of which was classified for training and the remaining 20% for testing, we trained 3754 subjects and tested the remining 938 subjects. A summary of the statistical analysis for the dataset is presented in Table 1.

Latitude and longitude are geographic coordinates that can be used to track the spread of COVID-19 across different regions and countries. By knowing the location of confirmed cases, health officials can identify potential hotspots and take targeted measures to contain the spread of the virus. Moreover, latitude and longitude information can be used with other demographic and environmental data to better understand the factors contributing to the spread of COVID-19, such as population density, air pollution levels, and weather patterns [50].

Total confirmed cases and cured/discharged/migrated data are important variables that can be used to track the progression of COVID-19 in a given region. By analyzing the number of confirmed cases over time, health officials can monitor the spread of the virus and make informed decisions about public health interventions. Similarly, the number of people who have been cured or discharged can provide insights into the effectiveness of treatments and the population’s overall health status.

Regarding COVID-19 mortality prediction, confirmed cases and cured/discharged/migrated data can help provide context and inform the development of predictive models. For example, the number of confirmed cases can be a useful predictor of mortality, as regions with a high number of confirmed cases can have a higher mortality rate due to the strain on healthcare resources. Similarly, regions with a high number of cured or discharged cases can have a lower mortality rate due to the effectiveness of treatments.

**Figure 3:**
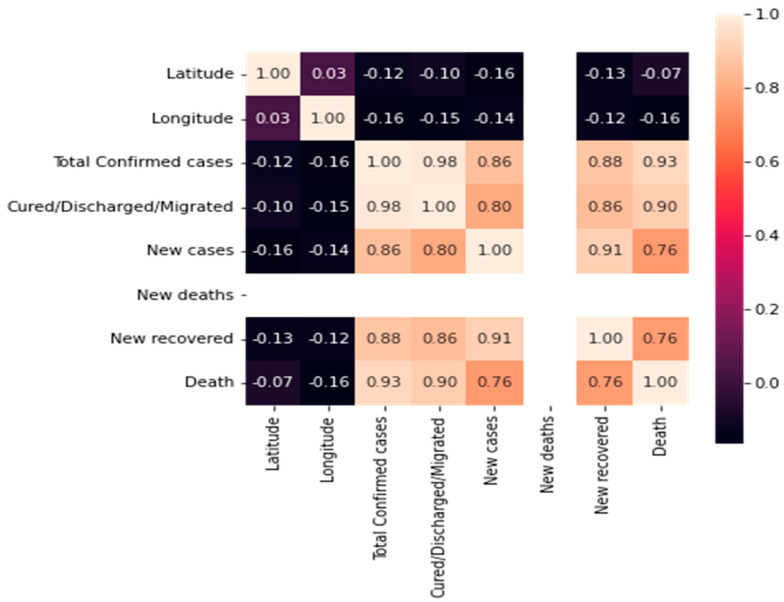
Heatmap matrix for the features.

A heatmap analysis can be a useful tool for exploring the relationships between different features in a COVID-19 mortality prediction dataset. By identifying correlations and potential confounding variables, heatmap analysis can help to inform the development of more accurate and robust predictive models. The heatmap analysis for the dataset features is shown in Figure 3. 

A box plot can be used to identify the distribution of the various features that are used as predictors in the predictive model. For example, a box plot can show the distribution of the number of confirmed cases, new deaths, or age, and highlight any potential outliers or anomalies that can affect the predictive model’s accuracy.

Box plots can also help compare the distributions of different features in the dataset. For example, a box plot can show the distribution of the number of deaths in different regions or populations, or compare the distributions of the number of confirmed cases and the number of new cases.

Histograms can be used to analyze the distribution of different features that can be important predictors of mortality. For example, a histogram can show the distribution of the number of confirmed cases, new deaths, or age, and help to identify any patterns or trends in the data.

**Figure 4:**
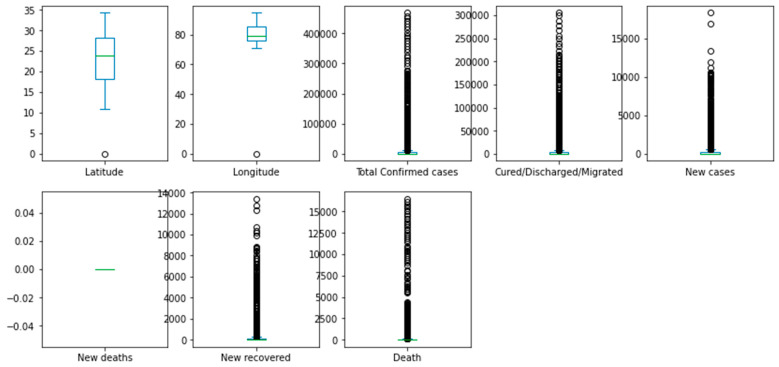
Box plot for the distribution analysis of the features.

**Figure 5:**
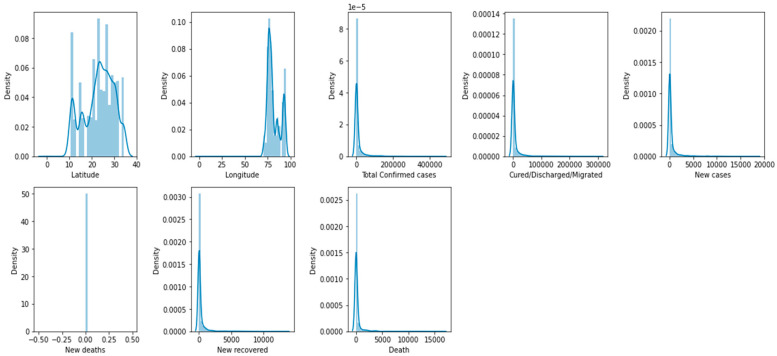
Histogram for the distribution analysis of the features.

Histograms are helpful in identifying the shape of the distribution of a feature, including whether it is symmetric, skewed, or has multiple peaks. A histogram shows the distribution of the number of deaths in different regions or populations, or compares the distributions of confirmed cases and new cases. Figure 4 demonstrates a box plot for the distribution analysis of the features. Figure 5 demonstrates the histogram for the distribution analysis of the features.

**Figure 6:**
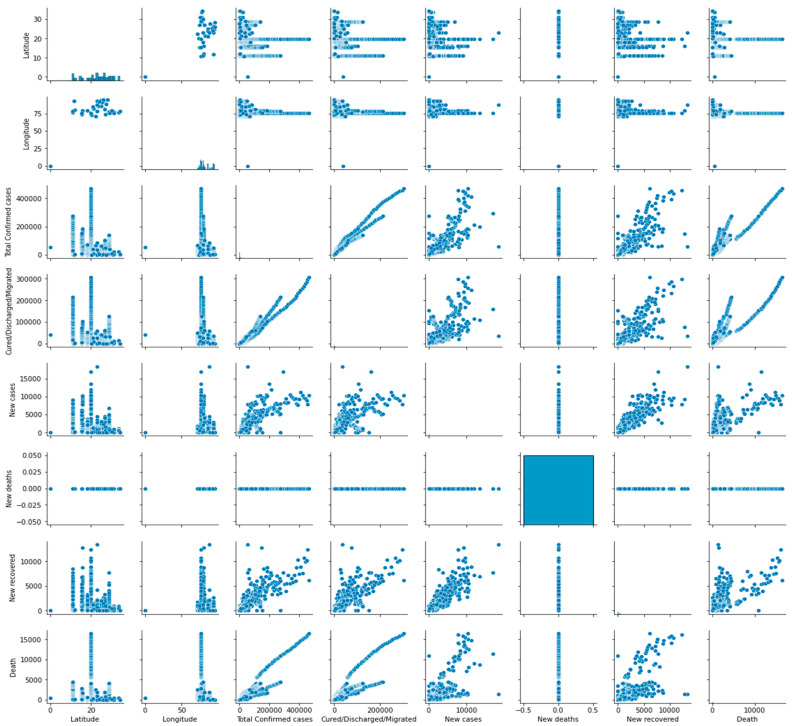
Pair plot for the distribution analysis of the features.

Pair plots are a valuable tool for detecting patterns and trends within a dataset and investigating potential associations between different features. They can aid in detecting non-linear relationships and correlations between features that are not immediately evident from the individual analysis. Figure 6 illustrates the pair plot utilized to analyze the distribution of the features.

## 5. Performance Indicators

Different models are compared using many validation techniques in this study, including MSE, MAE, MedAE, RMSE, and R^2^ [51].

### 5.1. Mean Squared Error (MSE)

The mean squared error (MSE) of an estimator is the average difference (in squares) between the estimated and actual values. The mean squared error approximates an estimator. It is always positive, and features that get closer to zero are preferable. MSE is calculated according to Equation (10).

(10) $$\mathrm{MSE}=\frac{1}{n}\sum_{t=1}^{n}{\left(Z_{t}-\hat{Z_{t}}\right)}^{2}$$

where *n* stands for the total number of data points, $Z_{t}$ indicates the observed value at time *t*, and *Ẑ_t_* represents the predicted value at time *t*.

### 5.2. Mean Absolute Error (MAE)

Without considering the direction of the forecasts, it assesses the average size of the inaccuracies. It is the norm over the test of the obvious differences between anticipation and true experience, where each different feature is given an equal weight. According to Equation (11), the acquired value of MAE is computed.

(11) $$\mathrm{MAE}\;\;\frac{\sum_{t=1}^{n}\left|r_{q}-r\right|}{n}$$

where *n* denotes the errors number, and $r_{q}-r$ stands for the absolute errors.

### 5.3. Median Absolute Error (MedAE)

According to Equation (12), this measure is the median of the absolute differences |$Z_{t}-\hat{Z_{t}}$| for every *n* pair of predictions and measurements.

(12) $$\mathrm{MedAE}\;=\;median\;(\left|Z_{1}-\hat{Z_{1}}\right|,\;\ldots ,\;\left|Z_{n}-\hat{Z_{n}}\right|)$$

### 5.4. Root Mean Squared Error (RMSE)

It is the residuals’ standard deviation. The percentage of residuals indicates how far away from the relapse line the information is focused. RMSE measures the degree of dispersion of these residuals. In a metaphorical sense, it reveals how evenly distributed the data are along the line of best fit. In climatology, forecasting, and relapse investigation, RMSE is frequently used to validate test results. Equation (13) contains the RMSE calculation formula.

(13) $$RMSE=\sqrt{\frac{1}{K}\sum_{M=1}^{k}{\left(a_{m}-\hat{a_{m}}\right)}^{2}}$$

where *k* denotes the number of observations, $a_{m}$ stands for the observed value, and $\hat{a_{m}}$ represents the predicted value.

### 5.5. Coefficient of Determination (R^2^)

It is an objective measure of how closely the data resemble the fitted relapse line. It is also known as the assurance coefficient or the assurance coefficient for repeated relapses. It is always in the range between 0% and 100%. 0% indicates that the model makes no distinction between the information about the changeability of the response around its mean, and 100% indicates that it makes no distinction between the information about the fluctuation of the reaction around its mean. Equation (14) provides the R^2^ mathematical equation.

(14) $$R^{2}\;=\;1-\frac{\sum {\left(t_{i}-\hat{t_{i}}\right)}^{2}}{{\left(t_{i}-\bar{t_{i}}\right)}^{2}}$$

where $t_{i}$ denotes the actual cumulative confirmed instances, $\hat{t_{i}}$ represents the anticipated cumulative confirmed cases, and $\bar{t_{i}}$ stands for the average of actual cumulative confirmed cases.

## 6. Experimental Results

The experiment results were obtained using Jupyter Notebook version 6.4.6, a widely used software tool for analyzing and visualizing data in the Python programming language. With Jupyter Notebook, users can write and execute code, create visualizations, and document their analysis within a single interface accessible via a web browser. The experiment was conducted on a computer running Microsoft Windows 10 and equipped with an Intel Core i5 processor and 16 GB of RAM.

In order to evaluate the performance of the proposed model (CNN-GRU) for predicting the COVID-19 death rate, six classification models, namely, Random Forest (RF) Regressor, Support Vector Regressor (SVR), K-Nearest Neighbor (KNN) Regressor, Bayesian Ridge (BR) Regressor, Gradient Boosting (GB) Regressor, and Dummy Regressor (DR) were used for comparison using the same dataset. The performance of these classification models was evaluated using MSE, MAE, RMSE, MedAE, and R^2^ metrics.

The CNN-GRU model consists of two convolution layers, one max-pooling layer, one GRU layer, one hidden layer, and an output layer that returns a single, continuous value. The first convolution layer consists of 32 filters, and the kernel size is 5. The second convolution layer consists of 16 filters, and the kernel size is 3. The GRU layer includes 100 hidden units. The hidden layer includes 16 neurons. The activation function used in the output layer is the linear function. The number of epochs used is 50. 

Table 2 displays the hyperparameters used for the regression models in the experiment. The Random Forest (RF) model was set with a value of 20 for its N_estimators parameter. In the K-Nearest Neighbor (KNN) regressor model, its N_neighbors are set to 10 and a weight function of “distance”. The Support Vector Regression (SVR) model was set with a tolerance value of 0.01, regularization value (C) of 1, and kernel set to “rbf” which means radial basis function (RBF) kernel SVM. The Gradient Boosting (GB) regressor model was set with a learning rate of 0.1, 200 estimators, and a maximum depth of 3. The Dummy Regressor (DR) model was set to use the “mean” strategy. The Bayesian Ridge (BR) regressor model was set to run for 300 iterations with a tolerance value of 0.001. Finally, the CNN hyperparameters are also mentioned in detail.

The reasons behind selecting these specific sets of hyperparameters for each model are as follows:

1. Random Forest (RF): N_estimators = 20: The number of estimators determines the number of decision trees in the random forest ensemble. A higher number of estimators can improve performance, but it also increases computational complexity. The value of 20 was likely chosen as a trade-off between accuracy and computational efficiency.
2. K-Nearest Neighbors (KNN): N_neighbors = 10: This parameter specifies the number of neighbors to consider for classification or regression. Choosing 10 neighbors suggests that the model should consider a relatively large neighborhood for making predictions. The weights = “distance” determines the weight assigned to each neighbor during prediction. By setting it to “distance,” the model gives higher weight to closer neighbors, which can be useful when the distribution of data points is uneven.
3. Support Vector Regression (SVR): Tol = 0.01: This parameter represents the tolerance for stopping criteria. A smaller tolerance value can lead to a more precise solution at the cost of increased computation time. C = 1: The C parameter controls the trade-off between achieving a smaller training error and a larger margin. A smaller C value allows more errors in the training set but may result in a wider margin. Kernel = “rbf”: The kernel parameter specifies the type of kernel function to be used. “Rbf” stands for radial basis function, which is commonly used for non-linear regression problems.
4. Gradient Boosting (GB): Learning_rate = 0.1: This parameter determines the step size at each boosting iteration. A smaller learning rate can make the model converge more slowly but may lead to better generalization. n_estimators = 200: The number of boosting stages to perform. Increasing the number of estimators can improve the model’s performance, but it also increases the computational cost. max_depth = 3: This parameter sets the maximum depth of each decision tree in the gradient boosting ensemble. Limiting the depth can prevent overfitting and promote better generalization.
5. Decision Tree Regression (DR): Strategy = “mean”: This parameter specifies the strategy to use when a node in the decision tree has no samples. The “mean” strategy replaces the missing value with the mean of the target values of the samples in that node.
6. Bayesian Ridge (BR): N_iter = 300: The number of iterations for the Bayesian Ridge estimator. Increasing the number of iterations allows the model to refine its estimates further. tol = 0.001: This parameter sets the tolerance for convergence. A smaller tolerance value indicates a more precise convergence criterion.

The selection of these hyperparameters depends on various factors, including the nature of the dataset, problem complexity, and computational constraints. The chosen hyperparameters aim to strike a balance between model performance and efficiency based on prior knowledge and experimentation. It is worth noting that hyperparameter tuning is often an iterative process, and the optimal values can vary depending on the specific problem and data.

Table 3 summarizes the performance of the regression models and the proposed CNN-GRU model. The Random Forest (RF) model had an MSE of 3.2 × 10^−5^, MAE of 0.003, MedAE of 0.0017, RMSE of 0.0057, and R^2^ of 0.64. The K-Nearest Neighbors (KNN) model had an MSE of 3.7 × 10^−5^, MAE of 0.004, MedAE of 0.0023, RMSE of 0.0060, and R^2^ of 0.60. The Support Vector Regressor (SVR) model had an MSE of 1.8 × 10^−6^, MAE of 0.001, MedAE of 0.0008, RMSE of 0.0013, and R^2^ of 0.96. The Gradient Boosting (GB) model had an MSE of 4.2 × 10^−5,^ MAE of 0.004, MedAE of 0.0028, RMSE of 0.0065, and R^2^ of 0.53. The Dummy Regressor (DR) model had an MSE of 7.06 × 10^−5^, MAE of 0.006, MedAE of 0.0043, RMSE of 0.0084, and R^2^ of 0.23. The Bayesian Ridge (BR) model had an MSE of 7.4 × 10^−5^, MAE of 0.006, MedAE of 0.0045, RMSE of 0.0085, and R^2^ of 0.22. The proposed CNN-GRU model achieved the best results; it had an MSE of 1.14 × 10^−9^, MAE of 2.5 × 10^−5^, MedAE of 1.8 × 10^−5^, RMSE of 3.3 × 10^−5^, and R^2^ of 0.99.

**Figure 7:**
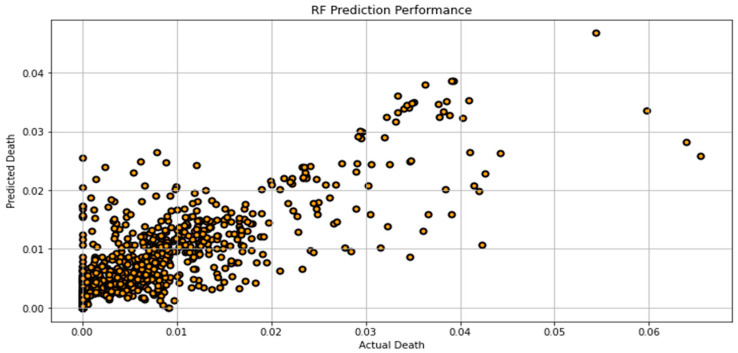
Actual death vs. predicted death for RF model performance.

**Figure 8:**
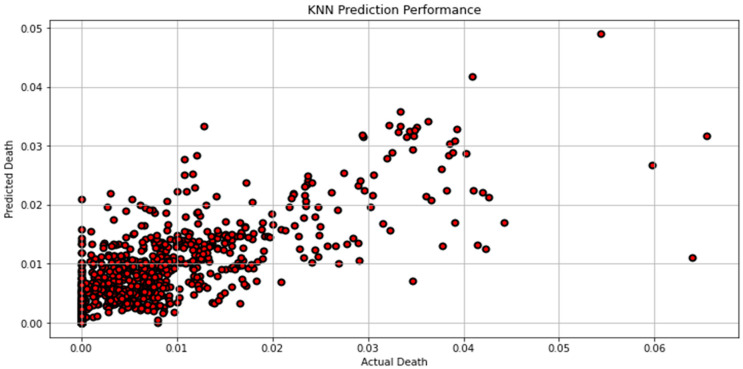
Actual death vs. predicted death for KNN model performance.

**Figure 9:**
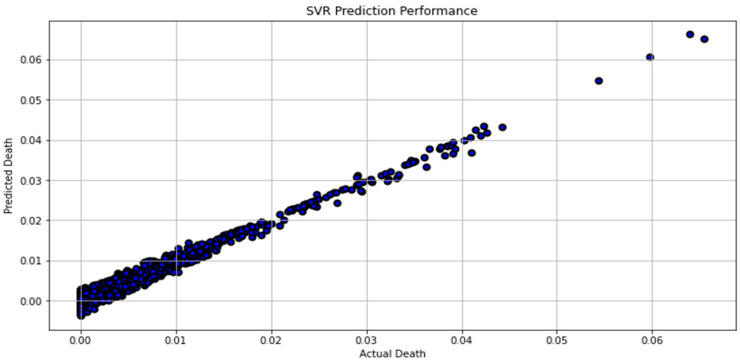
Actual death vs. predicted death for SVR model performance.

**Figure 10:**
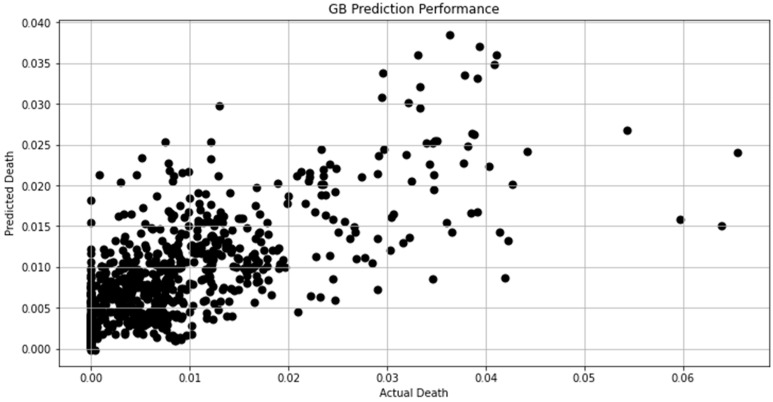
Actual death vs. predicted death for GB model performance.

**Figure 11:**
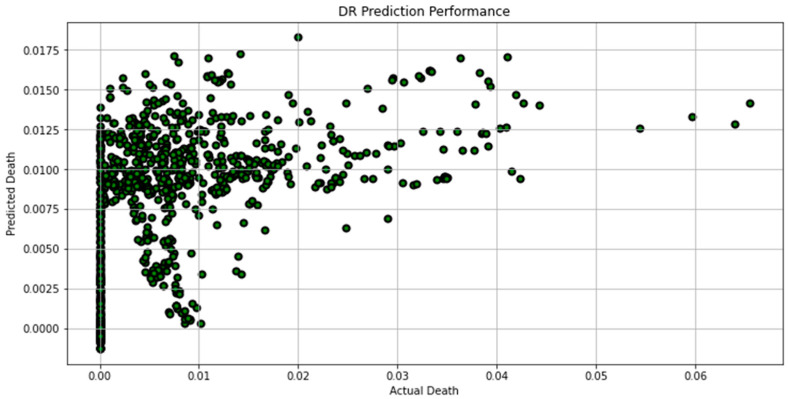
Actual death vs. predicted death for DR model performance.

**Figure 12:**
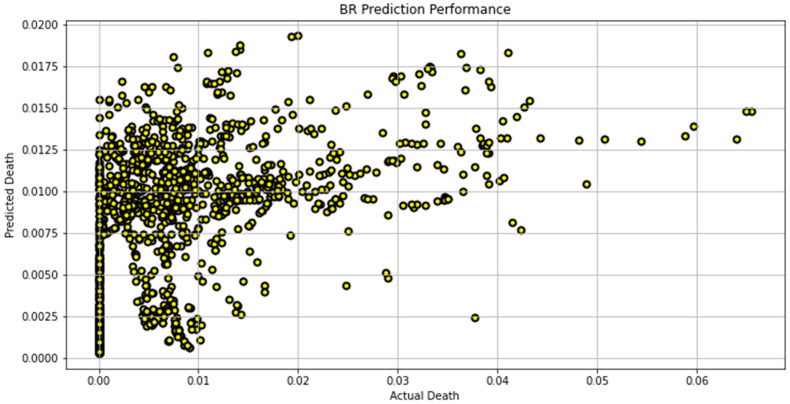
Actual death vs. predicted death for BR model performance.

**Figure 13:**
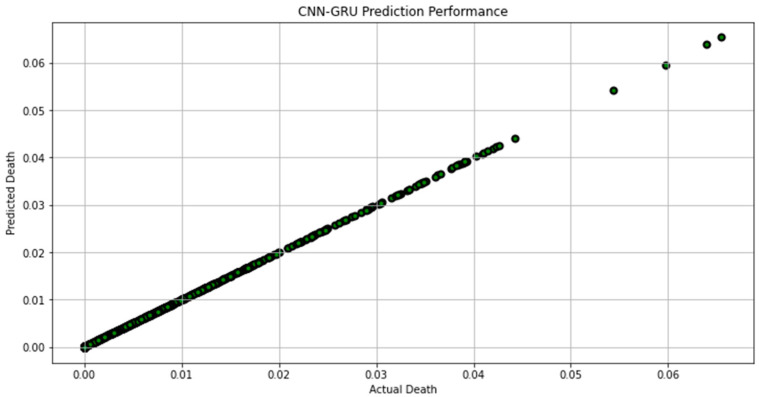
Actual death vs. predicted death for CNN-GRU model performance.

**Figure 14:**
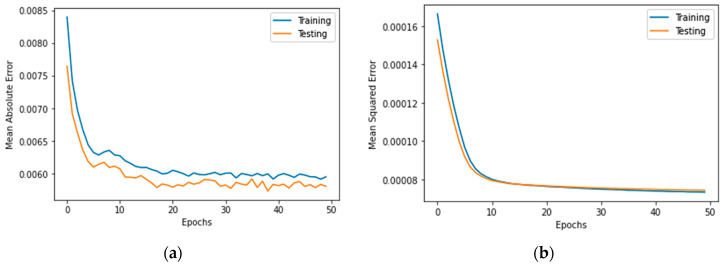
(**a**) Mean absolute error, and (**b**) mean squared error for 50 epochs using CNN-GRU model.

Figure 7, Figure 8, Figure 9, Figure 10, Figure 11, Figure 12 and Figure 13 demonstrate the actual death vs. predicted death for the models, namely, RF, KNN, SVR, GB, DR, BR, and the proposed CNN-GRU, respectively. Figure 14 displays the mean squared error and mean absolute error vs. the number of epochs using the CNN-GRU model.

RMSE is used to evaluate CNN-GRU model outcomes. Ten independent runs describe the CNN-GRU model in Table 4. These runs’ minimum, median, maximum, and mean average errors are provided. These error measurements from numerous models runs allow the evaluation of the CNN-GRU model’s consistency and efficiency. This detailed explanation facilitates the analysis of the model’s performance and reliability. 

Table 5 shows the ANOVA findings for the proposed CNN-GRU model and the other models. This statistical analysis seeks to explain model differences. ANOVA findings can show if model performance varies statistically. The Wilcoxon signed-rank test compares the CNN-GRU model and the other models in Table 6. This non-parametric test compares matched data, like model performance on the same dataset. The Wilcoxon signed-rank test and ten separate iterations of each model enable accurate comparisons and increase the study’s reliability. These statistical tests objectively evaluate the CNN-GRU model compared to the other models. ANOVA and Wilcoxon signed-rank *p*-values can be used to determine if model performance differences are statistically significant. These findings help explain the CNN-GRU model’s comparative efficacy and applicability for the job or dataset.

**Figure 15:**
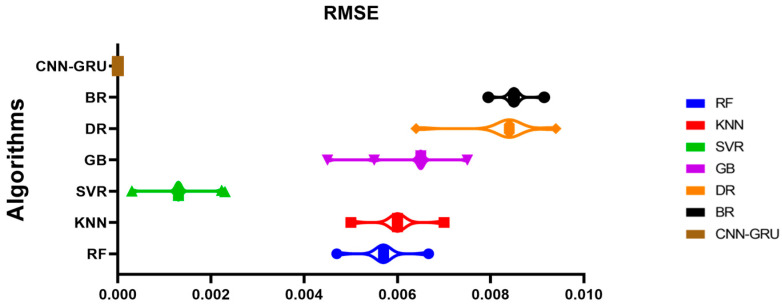
Plot of the presented CNN-GRU model and other models based on RMSE.

**Figure 16:**
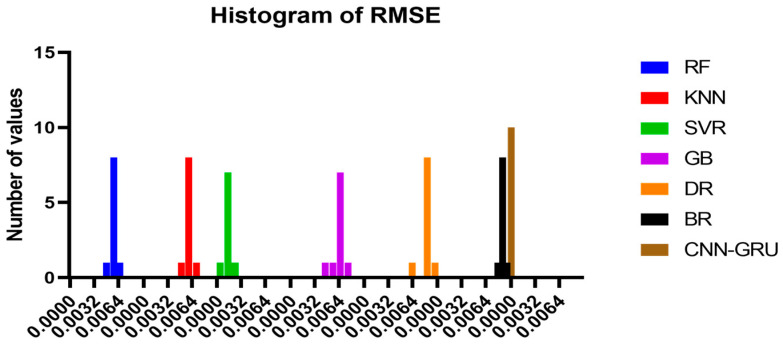
Histogram of RMSE for the presented CNN-GRU model and other models.

**Figure 17:**
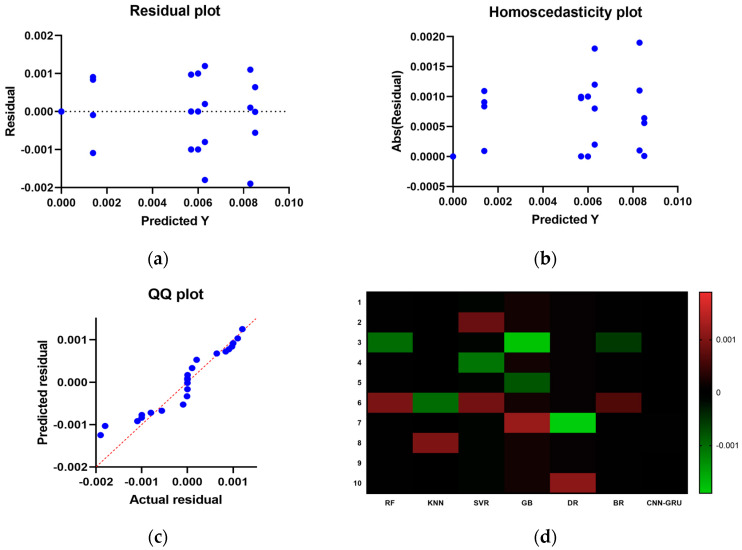
Compare and display the presented CNN-GRU model and other models. (**a**) residual plot, (**b**) homoscedasticity plot, (**c**) QQ plot, and (**d**) heat maps.

Figure 15 compares the CNN-GRU model to several RMSE-based models. Each model’s RMSE would be plotted to compare performance. Figure 16 shows the RMSE histograms for the proposed CNN-GRU model and other models. The histogram of RMSE values shows the spread and concentration of each model’s results. Figure 17 shows the QQ plots, residual plots, and heat maps of the proposed CNN-GRU model and other models. QQ plots compare error distributions to theoretical distributions. Residual plots show the model’s performance by comparing observed and anticipated values. The heat maps reveal data trends and correlations. These data show the CNN-GRU model’s capacity to outperform the other models in RMSE and provide a complete study of the model’s performance and attributes. To show the proposed CNN-GRU model advances, another dataset is compiled in Appendix A.

## 7. Conclusions and Perspectives

The COVID-19 pandemic has presented a global challenge transcending boundaries, such as provincial, radical, conceptual, spiritual, social, and pedagogical. To effectively monitor COVID-19 patients, an interconnected network facilitated by the Internet of Things (IoT) is useful. This technology enables patients to identify symptoms and receive treatment quickly, and artificial intelligence (AI) is used to measure, assess, and diagnose the risk of infection. In addition, AI can also predict the number of alternate incidents, recoveries, and casualties, and forecast future cases. IoT technologies have also been employed to minimize COVID-19 exposure to others through prenatal screening, patient monitoring, and post-patient incident response procedures. This study utilized an advanced CNN-GRU model with data normalization and imputation for COVID-19 death prediction using an Indian dataset that contained eight features and 4692 instances. Five evaluation metrics were used to assess the performance of the model, and the results showed that the CNN-GRU model outperformed other models for COVID-19 death prediction achieving the lowest values of MSE, MAE, MedAE, and RMSE, and the highest R^2^. Thus, the CNN-GRU model is recommended for similar predictive tasks. There are several possible avenues for future work in this area:

1. Incorporate more data sources: Many potential data sources could be used to improve COVID-19 death prediction models. For example, social media data could be used to track the spread of misinformation about COVID-19, which could help identify regions at a higher risk of experiencing a surge in cases and deaths. Additionally, data on weather patterns, pollution levels, and other environmental factors could be integrated to better understand how these variables affect the spread and severity of COVID-19.
2. Improve model accuracy: There are several ways to improve the accuracy of COVID-19 death prediction models. One approach is to use more sophisticated machine learning algorithms, such as reinforcement learning, which can handle more complex data and provide more accurate predictions. Another method is to improve the data quality used to train the models, for example, by incorporating more granular data on individual patients’ health status and medical history.
3. Develop models for specific populations: COVID-19 has been shown to affect different populations in different ways, with some people (such as the elderly or those with underlying health conditions) at a higher risk of death than others. Therefore, developing targeted models for specific populations could be an effective way to improve the accuracy of COVID-19 death predictions and better allocate resources for prevention and treatment.
4. Combine prediction models with interventions: Predicting COVID-19 death is only helpful if the information can be used to take action to prevent deaths. Therefore, future work could integrate death prediction models with intervention strategies, such as targeted vaccination campaigns, lockdown measures, or specific treatments for high-risk patients. By combining prediction models with interventions, public health officials could take a more proactive approach to managing the pandemic and reducing the number of COVID-19 deaths.

## Figures and Tables

**Table 1:** Statistical calculation for the features.

	Count	Mean	Std.	Min.	50%	Max.
Latitude	4692	23.185	6.6359	0.0	23.9408	34.2996
Longitude	4692	81.451	6.9594	0.0	79.0193	94.7278
Total confirmed cases	4692	11,393	37,208	1.0	619.0000	468,265
Cured/Discharged/Migrated	4692	6908	23,390	0.0	197.5000	305,521
New cases	4692	418.6	1259	0.0	26.0000	18,366
New deaths	4692	0.0	0.0	0.0	0.0	0.0
New recovered	4692	283.06	947.9	−1.0	8.0000	13,401
Death	4692	291.28	1213	0.0	5.0000	16,476

**Table 2:** Hyperparameters for the regression models.

Models	Hyperparameters
RF	N_estimators = 20.
KNN	N_neighbors = 10, weights = “distance”.
SVR	Tol = 0.01, C = 1, kernel = “rbf”.
GB	Learning_rate = 0.1, n_estimators = 200, max_depth = 3.
DR	Strategy = “mean”.
BR	N_iter = 300, tol = 0.001.
CNN	learnRate = 0.001, hiddenLayerTwo = 256, hiddenLayerOne = 256, epochs = 40, dropout = 0.4, batch_size = 32

**Table 3:** Performance of the regression models and the proposed CNN-GRU model.

Model	MSE	MAE	MedAE	RMSE	R^2^
RF	3.2 × 10^−5^	0.003	0.0017	0.0057	0.64
KNN	3.7 × 10^−5^	0.004	0.0023	0.0060	0.60
SVR	1.8 × 10^−6^	0.001	0.0008	0.0013	0.96
GB	4.2 × 10^−5^	0.004	0.0028	0.0065	0.53
DR	7.06 × 10^−5^	0.006	0.0043	0.0084	0.23
BR	7.4 × 10^−5^	0.006	0.0045	0.0085	0.22
CNN-GRU	1.14 × 10^−9^	2.5 × 10^−5^	1.8 × 10^−5^	3.3 × 10^−5^	0.99

**Table 4:** A description of the proposed CNN-GRU model as well as the results of other models based on the RMSE factor.

	RF	KNN	SVR	GB	DR	BR	CNN-GRU
Number of values	10	10	10	10	10	10	10
Minimum	0.0047	0.005	0.0003	0.0045	0.0064	0.00795	3.3 × 10^−6^
25% Percentile	0.0057	0.006	0.0013	0.00625	0.0084	0.0085	3.3 × 10^−6^
Median	0.0057	0.006	0.0013	0.0065	0.0084	0.0085	3.3 × 10^−6^
75% Percentile	0.0057	0.006	0.001533	0.0065	0.0084	0.0085	3.33 × 10^−6^
Maximum	0.00667	0.007	0.0023	0.0075	0.0094	0.00915	3.5 × 10^−6^
Range	0.00197	0.002	0.002	0.003	0.003	0.0012	2 × 10^−7^
Mean	0.005697	0.006	0.001393	0.0063	0.0083	0.00851	3.33 × 10^−6^
Std. Deviation	0.000464	0.000471	0.000556	0.000789	0.000738	0.000284	6.75 × 10^−8^
Std. Error of Mean	0.000147	0.000149	0.000176	0.000249	0.000233	8.97 × 10^−5^	2.13 × 10^−8^
Sum	0.05697	0.06	0.01393	0.063	0.083	0.0851	3.33 × 10^−5^

**Table 5:** The ANOVA test for the presented CNN-GRU model and other models.

	SS	DF	MS	F (DFn, DFd)	*p* Value
Treatment (between columns)	0.000642	6	0.000107	F (6, 63) = 375.4	*p* < 0.0001
Residual (within columns)	1.79 × 10^−5^	63	2.85 × 10^−7^	-	-
Total	0.00066	69	-	-	-

**Table 6:** The Wilcoxon signed-rank test for the presented CNN-GRU model and other models.

	RF	KNN	SVR	GB	DR	BR	CNN-GRU
Theoretical median	0	0	0	0	0	0	0
Actual median	0.0057	0.006	0.0013	0.0065	0.0084	0.0085	3.3 × 10^−6^
Number of values	10	10	10	10	10	10	10
Wilcoxon signed-rank test							
Sum of signed ranks (W)	55	55	55	55	55	55	55
Sum of positive ranks	55	55	55	55	55	55	55
Sum of negative ranks	0	0	0	0	0	0	0
*p* value (two-tailed)	0.002	0.002	0.002	0.002	0.002	0.002	0.002
Exact or estimate?	Exact	Exact	Exact	Exact	Exact	Exact	Exact
Significant (alpha = 0.05)?	Yes	Yes	Yes	Yes	Yes	Yes	Yes
How big is the discrepancy?							
Discrepancy	0.0057	0.006	0.0013	0.0065	0.0084	0.0085	3.3 × 10^−6^

**Figure A1:**
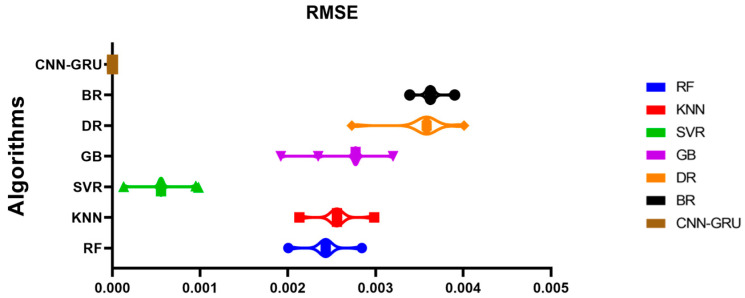
Graph depicting the showcased CNN-GRU model and alternative models with respect to the RMSE.

**Figure A2:**
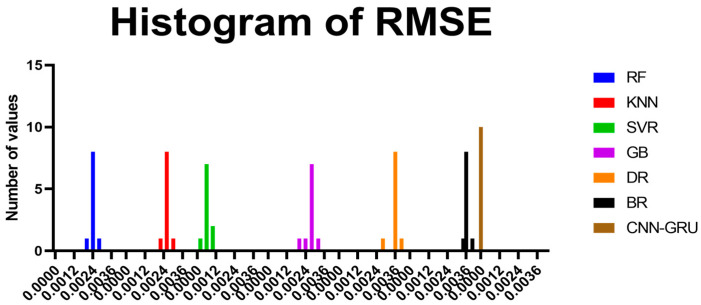
RMSE distribution chart for the featured CNN-GRU model and alternative models.

**Figure A3:**
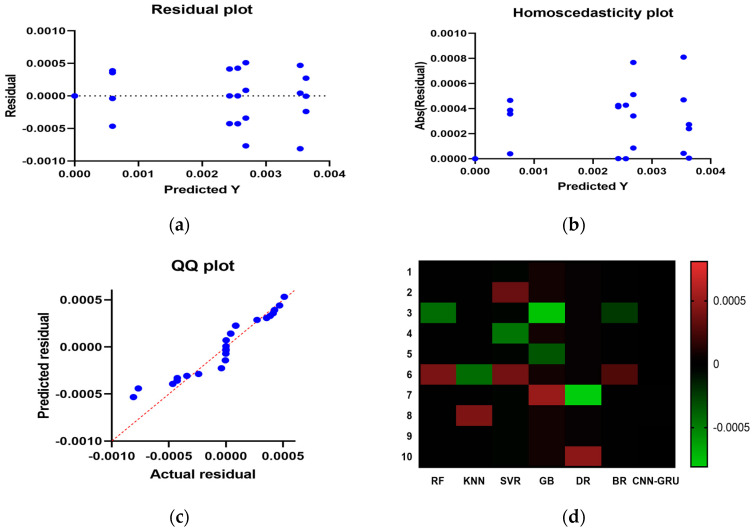
Visualize and compare the supplied CNN-GRU model to others. (**a**) residual plot, (**b**) homoscedasticity plot, (**c**) QQ plot, and (**d**) heat maps.

## Data Availability

These datasets have been sourced from publicly accessible databases, specifically from the URLs https://www.kaggle.com/datasets/imdevskp/covid19-corona-virus-india-dataset, (accessed on 6 November 2023) and https://www.kaggle.com/datasets/imdevskp/corona-virus-report, (accessed on 19 June 2023).

## Appendix A

To show the proposed CNN-GRU model advances, another dataset is compiled, which is available at https://www.kaggle.com/datasets/imdevskp/corona-virus-report, (accessed on 6 November 2023).

The CNN-GRU model outcomes are assessed using RMSE. Table A1 presents the description of the CNN-GRU model based on ten independent runs, showcasing the minimum, median, maximum, and mean average errors. These runs’ minimum, median, maximum, and mean average errors are provided. By considering error measurements across multiple model runs, one can thoroughly evaluate the consistency and efficiency of the CNN-GRU model. This comprehensive explanation facilitates the analysis of the model’s performance and reliability.

Table A2 presents the ANOVA results for both the CNN-GRU model and the comparative model. This statistical analysis aims to elucidate any distinctions between the models. ANOVA findings are instrumental in revealing whether there are statistically significant variations in model performance. The Wilcoxon Signed Rank test, as delineated in Table A3, compares the CNN-GRU model with the models under comparison. Conducted over ten separate iterations for each model, the Wilcoxon Signed-Rank test ensures precision in comparisons and enhances the overall reliability of the study. These statistical assessments provide an objective evaluation of the CNN-GRU model in relation to other models. By examining the p-values from ANOVA and the Wilcoxon Signed-Rank test, one can ascertain whether the observed differences in model performance are statistically significant. Such findings contribute to a better understanding of the CNN-GRU model’s relative effectiveness and suitability for a given task or dataset.

**Table A1:** An overview of the proposed CNN-GRU model and an examination of the outcomes of alternative models, focusing on the RMSE metric.

	RF	KNN	SVR	GB	DR	BR	CNN-GRU
Number of values	10	10	10	10	10	10	10
Minimum	0.002004	0.002132	0.000128	0.001919	0.002729	0.00339	1.41 × 10^−6^
25% Percentile	0.002431	0.002559	0.000554	0.002665	0.003582	0.003625	1.41 × 10^−6^
Median	0.002431	0.002559	0.000554	0.002772	0.003582	0.003625	1.41 × 10^−6^
75% Percentile	0.002431	0.002559	0.000653	0.002772	0.003582	0.003625	1.42 × 10^−6^
Maximum	0.002844	0.002985	0.000981	0.003198	0.004009	0.003902	1.49 × 10^−6^
Range	0.00084	0.000853	0.000853	0.001279	0.00128	0.000512	8 × 10^−8^
Mean	0.00243	0.002559	0.000594	0.002687	0.003539	0.003629	1.42 × 10^−6^
Std. Deviation	0.000198	0.000201	0.000237	0.000336	0.000315	0.000121	2.7 × 10^−8^
Std. Error of Mean	6.26 × 10^−5^	6.36 × 10^−5^	7.5 × 10^−5^	0.000106	9.95 × 10^−5^	3.83 × 10^−5^	8.54 × 10^−9^
Sum	0.0243	0.02559	0.005938	0.02687	0.03539	0.03629	1.42 × 10^−5^

**Table A2:** The ANOVA analysis for the provided CNN-GRU model and alternative models.

ANOVA Table	SS	DF	MS	F (DFn, DFd)	*p* Value
Treatment (between columns)	0.000117	6	1.95 × 10^−5^	F (6, 63) = 375.3	*p* < 0.0001
Residual (within columns)	3.26 × 10^−6^	63	5.18 × 10^−8^		
Total	0.00012	69			

**Table A3:** The Wilcoxon Signed-Rank test applied to the showcased CNN-GRU model and alternative models.

	RF	KNN	SVR	GB	DR	BR	CNN-GRU
Theoretical median	0	0	0	0	0	0	0
Actual median	0.002431	0.002559	0.000554	0.002772	0.003582	0.003625	1.41 × 10^−6^
Number of values	10	10	10	10	10	10	10
Wilcoxon Signed Rank Test							
Sum of signed ranks (W)	55	55	55	55	55	55	55
Sum of positive ranks	55	55	55	55	55	55	55
Sum of negative ranks	0	0	0	0	0	0	0
*p* value (two tailed)	0.002	0.002	0.002	0.002	0.002	0.002	0.002
Exact or estimate?	Exact	Exact	Exact	Exact	Exact	Exact	Exact
Significant (alpha = 0.05)?	Yes	Yes	Yes	Yes	Yes	Yes	Yes
How big is the discrepancy?							
Discrepancy	0.002431	0.002559	0.000554	0.002772	0.003582	0.003625	1.41 × 10^−6^

Figure A1 illustrates a comparison between the CNN-GRU model and various models based on RMSE. The RMSE for each model is plotted to facilitate a performance comparison. In Figure A2, histograms of RMSE for both the CNN-GRU model and comparative models are presented, visually depicting the distribution and concentration of RMSE values for each model. Figure A3 showcases QQ plots, residual plots, and heat maps for the CNN-GRU model and comparative models. QQ plots are used to assess the distribution of errors in comparison to theoretical distributions, while residual plots demonstrate the model’s performance by contrasting observed and expected values. The heat map provides insights into data trends and correlations. These visualizations collectively demonstrate the CNN-GRU model’s superior performance in RMSE compared to the models under consideration, offering a comprehensive analysis of the model’s attributes and overall effectiveness.
